# Patterns of malaria indices across three consecutive seasons in children in a highly endemic area of West Africa: a three times-repeated cross-sectional study

**DOI:** 10.1186/1475-2875-13-199

**Published:** 2014-05-28

**Authors:** Denis-Luc Ardiet, Bertrand Graz, Thomas Szeless, Anne Mauris, Jacques Falquet, Ogobara K Doumbo, Amagana Dolo, Ousmane Guindo, Mahamadou S Sissoko, Moussa Konaré, Sandrine Motamed, André C Rougemont

**Affiliations:** 1 IMSP: Institut de Médecine Sociale et Préventive (currently Institut de santé globale), CMU, rue Michel Servet 1, Genève 4 CH-1211, Switzerland; 2 Association Mali-Genève/Community Health - Geneva, Villa Friedheim, chemin de la Tour de Champel 17, CH-1206 Genève, Switzerland; 3 Malaria Research and Training Centre, Faculty of Medicine, Pharmacy and Odontostomatology, University of Sciences, Techniques and Technology, P.O. Box 1805, Bamako, Mali

**Keywords:** Plasmodium infection, Malaria disease, Season, Endemicity, Malaria indices, Epidemics, Epidemiology, Diagnosis

## Abstract

**Objectives:**

To study the manifestations of *Plasmodium* infection, and its relations with the malaria disease, especially when comparing dry and rainy seasons in a hyperendemic area of West Africa.

**Methods:**

The study was carried out in an area where malaria transmission is high, showing important seasonal variations. One thousand children, representing the total child population (1–12 year old), were observed transversally at the end of three consecutive seasons (dry/rainy/dry). The usual indicators, such as parasite density, splenomegaly, anaemia, or febrile disease were recorded and analysed.

**Results:**

The prevalence of *Plasmodium falciparum* was high in all age groups and seasons, constantly around 60%. The high transmission season (rainy) showed higher rates of anaemia and spleen enlargement and, in the youngest children only, higher parasite densities. There were also differences between the two dry seasons: in the first one, there was a higher rate of fever than in the second one (p < 0.001). Low parasite density (<2,000 p/μl) was *never* associated with fever during *any* season, raising some concern with regard to the usefulness of parasite detection. The possible origins of fever are discussed, together with the potential usefulness of analyzing these indices on a population sample, at a time when fever incidence rises and malaria is one potential cause among others. The distinction to be made between the *Plasmodium* infection and the malaria disease is highlighted.

**Conclusions:**

These data confirm previous hypotheses of a strong difference in malaria infection and disease between dry and rainy seasons. The most relevant seasonal indicator was not mainly parasite rate and density but anaemia, spleen enlargement, prevalence and possible origin of fever.

**Recommendations:**

In any situation (i.e. fever or not) and especially during the dry season, one must consider that detection of parasites in the blood is only evidence of a *Plasmodium* infection and not necessarily of a malaria disease. In such a situation, it seems suitable to obtain, through national malaria teams, a well-defined situation of transmission and prevalence of *Plasmodium* infection following zones and seasons, in order to adapt control strategies. For researchers, a systematic management of data separately for dry and rainy season appears mandatory.

## Background

In regions where malaria is highly prevalent, malaria-related morbidity and mortality are high and mainly affect subjects whose immunity is considered to be low, mostly young children (clinical signs going from simple febrile episodes to severe malaria with its complications) and pregnant women, especially during their first pregnancy [1,2]. Many factors may affect the pathophysiology of malaria: transmission intensity, prevalence of *Plasmodium* infection in the population, prevalence of other diseases, genetic background, age, season, or nutrition [3,4]. Because the relative importance of these factors changes from an area to another, malaria control strategies should require local/regional adaptations. In particular, the detection of malaria parasites cannot be a universal rule for diagnosis of the disease: blood parasite density, despite technical limits and physiological variations, can help estimating the prevalence of malaria infections in a population. However, where parasites are widespread (i.e. moderate to high endemicity), its usefulness as the “gold standard” for the diagnosis of the malaria disease is questionable. A previous work estimated that above a prevalence of 20% in the general population, the detection of parasites in the blood becomes useless for confirming that a febrile patient suffers from malaria [5]. To make an accurate diagnosis of the malaria disease, the use of rapid diagnostic tests (RDT) is equivalent to examination of blood films, since both methods have quite similar sensitivity and specificity [6]. Moreover in high transmission areas, the specificity of either technique is low to determine a malaria disease because the prevalence of *Plasmodium* infection is high [7].

Within a given area, there are often differences between the patterns of malaria indices between high and low-transmission seasons [8]: parasite density and parasite rate, rate of anaemia, or splenic rate are examples of such parameters that can vary over several months according to the transmission intensity. In addition, fever is not associated with the same symptoms at different periods of the year [9].

Among other signs, anaemia is strongly linked to the malaria disease in areas where malaria transmission occurs [10,11], and has been shown to be associated with season, young age, splenomegaly, parasite density, as well as other factors [9,12-15]. Decrease of haematocrit (or packed cell volume, PCV) and/or haemoglobin concentration also occurs in asymptomatic children both by hemolytic destruction, erythrocyte sequestration and bone marrow suppression [13,16]. Although anaemia (SA, severe anaemia; MA, moderate anaemia) is certainly one of the main consequences of malaria infection and disease, its role as a risk factor for fever incidence has also been suggested, even if this association has yet to be explained [9].

In order to explore further the manifestations of the malaria disease and the dynamics of malaria indices during dry and wet seasons in a hyperendemic area, about one thousand children were observed in a three times repeated cross-sectional study.

## Methods

### Study area and population

The study aimed at following most common malaria indices during three consecutive seasons in children from 1 to 12 years in the rural village of Mendela (20 km south-east of Sikasso, the third administrative Region of the Republic of Mali). The main criteria for the choice of the village were based on the need of a population size of about 1,000 children aged from one to 12 years of age and the absence of a permanent pond throughout the year.

The rainy season lasts six months (May – October) with a peak of precipitations (75% of 1100 mm annual rainfall) occurring from July to September. To check the possibility of an early malaria transmission during the dry seasons, daily rainfall records were obtained retrospectively from Sikasso meteorological station (latitude: 11.350° North; longitude: 5.683° West) through the U.S. National Climatic Data Center and the average daily rainfall was calculated on the period comprised between day 15 and day 45 prior to the medical and biological measurements [17]. Because the data from high-transmission seasons are generally more abundant and because the malaria pattern remains unclear during the low-transmission season, this survey was performed every six months at the end of two dry seasons and one rainy season (see Table 1).

**Table 1:** **Sex/age distributions (number and percentages) and parasite rates ( *P. falciparum *) of children included in the Mendela survey during the three seasons**

	**Dry season 2001**			**Rainy season 2001**			**Dry season 2002**		
	**n**	**%**	**Parasite rate (%)**	**n**	**%**	**Parasite rate (%)**	**n**	**%**	**Parasite rate (%)**
Sex									
F	365	51.5	57.5	361	51.4	56.5	358	49.2	59.0
M	344	48.5	58.4	341	48.6	60.1	370	50.8	57.0
Age									
0-1 year	4	0.6	75.0	2	0.3	100	8	1.1	50.0
1-3 years	119	16.8	52.1	90	12.8	66.7	110	15.1	45.5
3-6 years	230	32.4	60.0	223	31.8	59.6	208	28.6	63.0
6-9 years	197	27.8	58.4	200	28.5	53.5	181	24.9	63.5
9-12 years	144	20.3	57.6	158	22.5	60.1	155	21.3	56.8
12-15 years	15	2.1	66.6	29	4.1	48.3	66	9.1	50.0
Total	709	100	-	702	100	-	728	100	-

### Design

The study was cross-sectional with three surveys in the same village. A total of 709 children were observed during the first dry season, 702 at the end of the rainy season, and 728 at the end of the second dry season (Table 1): although 517 children were seen at each of the 3 cross-sectional survey, some differences can be observed in the participation of the subjects from one survey to another: because of logistical, individual, seasonal and cultural reasons it was not possible to fully control this.

Following acceptation of the study from National Ethical Committee, Regional Direction of Health and Village Authorities, the purpose of the study was presented to the villagers. The family head of each individual participant signed an informed consent form.

### Clinical and biological measurements

Axillary body temperature was measured using an electronic thermometer (Terumo®). Fever was defined as a temperature exceeding 37.5°C. PCV (packed cell volume or haematocrit) was chosen as the index to estimate anaemia, resulting from previous observations in the same region where PCV is strongly linked to haemoglobin concentration, leading mostly to normochromic anaemia. PCV was immediately assessed twice using a micro haematocrit centrifugator (Hettich®). According to WHO standards, severe anaemia (SA) was defined as PCV <30%. In this analysis, 32% was the threshold used for moderate anaemia (MA). Blood films were prepared from capillary blood and read by an experienced parasitologist from the Malaria Research and Training Centre, Faculty of Medicine, Pharmacy and Odontostomatology, University of Sciences, Techniques and Technology, P.O. Box 1805, Bamako, Mali, in order to estimate blood parasite density. The parasite count was calculated on the basis of an average concentration of 7,500 leukocyte/μl after observing 300 leukocytes in blood films. In the text, the different classes of parasite densities are referred as to: “very low” (1 to 199 p/μl), “low” (200 to 1,999 p/μl), “moderate” (2,000 to 19,999 p/μl) and “high” (20,000 p/μl or more). Splenomegaly was evaluated by palpation and classified according to the criteria of Hackett, by MDs from the national team.

### Statistical analyses

Analyses were performed using the R statistical software. The agreement between both PCV measurements was checked by the method described previously, analysing the differences of pairs of measurements against their means [18]. During the survey, 972 children between six months and 15 years of age were visited at least once, and 2,139 complete records (with age, body temperature, parasite density, splenomegaly and haematocrit properly registered) were obtained for the three seasons. Since the vast majority of children were between one and 12 years of age, inference statistics are presented only within this range when stratifying on age. Comparisons of percentages were made using Pearson’s chi-square test or Fisher’s exact test, and because malaria indices vary during childhood, their associations with fever were adjusted for age using bivariate logistic regression. Comparisons of parasite densities were achieved with Wilcoxon sum rank test. Because the distribution of parasite density is right-skewed, geometric means (GMPD) were calculated on the base 10 logarithms of individual values of positive subjects, and the calculated means were then back transformed. All p-values are indicated with a limit of 0.001, but they were much lower in numerous instances.

## Results

### Description of the population sample, prevalence of *P. falciparum* and precipitations

The average daily rainfall, calculated as described in the Methods section, was estimated at 1.6, 5.2 and 2.7 mm prior to the first dry season, rainy season and second dry season respectively. During the three surveys, 58% of children included in the study were aged between 3 and 9 years, while the children below 3 years of age represented about 16% of the sample. Females and males were equally represented during each survey (Table 1).

The prevalence of *Plasmodium malariae* varied between 3% and 12%, while *Plasmodium ovale* was detected marginally. In contrast, the prevalence of *P. falciparum* was high in all age groups and seasons, constantly around 60%. Only in younger children (aged 1–3 years), significant differences were observed in the prevalence of *P. falciparum*, between the rainy season and the dry seasons (p < 0.05).

### Seasonal variations of anaemia, splenomegaly and *P. falciparum* densities

**Figure 1:**
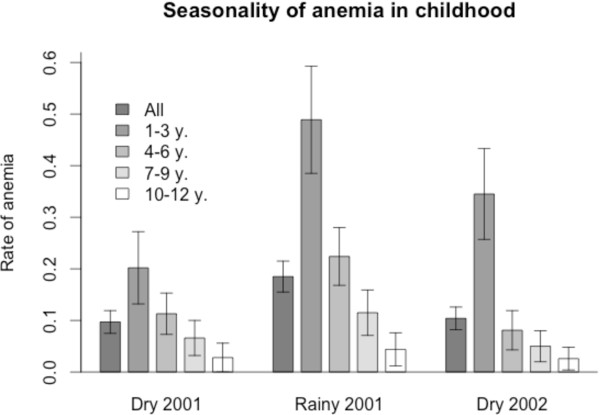
**Seasonal variations of severe anemia rates.** Bar charts representing the rate of severe anaemia (PCV < 30%) in different age classes over the three consecutive seasons. Two-sided error bars show 95% confidence intervals.

**Figure 2:**
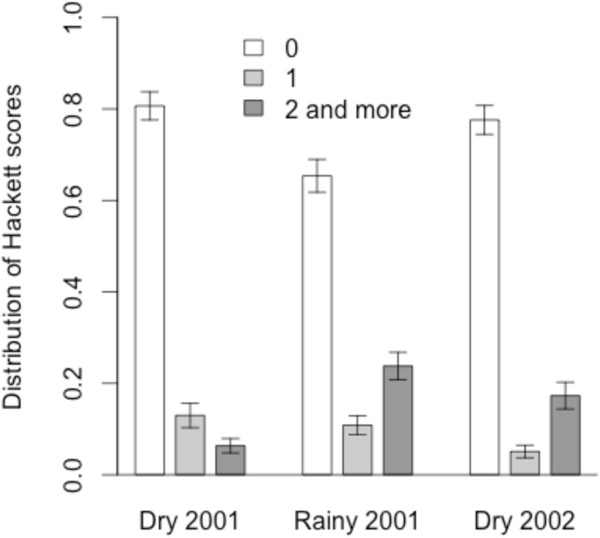
**Seasonal variations of splenomegaly.** Bar charts representing the distribution of spleen scores during each season. Error bars represent 95% confidence intervals.

The definition of mild and severe anaemia is based on cutoff values chosen from the distribution of haematocrit (PCV). The haematocrit level was subject to variations between age classes (anaemia decreases as the age increases, Figure 1). By far, the youngest children (1–3 y.) had higher rates of severe anaemia (p < 0.02, p < 0.001, p < 0.001 during the three consecutive seasons). The rate of severe anaemia was also linked with seasons, with a doubling of this rate during the rainy season: 9.7% (95% CI: 7.7-12.1) in the first dry season, 17.8% (95% CI: 15.2-20.8) in the rainy season, and 10.4% (95% CI: 8.3-13.0) in the second dry season (all children). Figure 2 displays the distribution of children according to the degree of splenomegaly: there was no difference in the spleen rate between the two dry seasons (p = 0.172), while it was significantly higher during the rainy season (p < 0.001). The magnitude of splenomegaly in the population of children at each seasonal follow-up was also assessed by comparing the means of spleen scores: this parameter was the lowest during the first dry season (mean = 0.26; SD = 0.58), the highest during the rainy season (mean = 0.60; SD = 0.88, p < 0.001) and intermediate during the second dry season (mean = 0.41; SD = 0.81, p < 0.05).

Additional file 1 shows mean PCV values and anaemia rates from different strata, highlighting the magnitude of anaemia in children. When stratifying on season, age group, and splenomegaly, PCV values from all strata were significantly different from the reference values (those from the first dry season, the youngest age class, and in the absence of spleen enlargement) and the calculation of odds ratios demonstrated increasing risks of anaemia among each level of these strata. This confirms previous findings that season, young age, and splenomegaly are linked with anaemia. Also note that severe anaemia was associated with splenomegaly (spleen score >1) during the first dry season (p < 0.001) and the rainy season (p < 0.05), but not during the second dry season (p = 0.784). When stratifying on parasite densities, a significant difference was observed between PCV means from children harbouring no parasites and those having moderate or high parasite density (p < 0.001). Similarly, only moderate and high parasite densities were significantly associated with an increase in severe anaemia and moderate anaemia rates. However, no significant difference in mean PCV values was observed with children harbouring low (p = 0.061) or very low parasite density (p = 0.781).

The geometric mean of *P. falciparum* density (GMPD) of positive subjects varied according to age group, season and haematocrit level (Table 2): GMPD was higher in children 1–3 years of age and decreased as the age increased; parasite density also increased in subjects with spleen enlargement. Finally, there was a higher GMPD in individuals having less than 32% haematocrit compared to children with higher haematocrit levels (p < 0.001).

**Table 2:** Parasite densities among different strata of children

**Variable**		**All children**	**Children positive for P. falciparum**			
	**Class**	**Prevalence**	**AMPD**	**GMPD**	**n**	**p-value (‡)**
Season	Dry 2001	58.0%	6143	1226	411	-
	Rainy 2001	58.3%	5246	1151	409	0.934
	Dry 2002	57.8%	3342	401	421	<0.001**
Age group	1-3 years	53.9%	12630	1685	172	-
	4-6 years	60.8%	4461	1101	402	0.019*
	7-9 years	58.3%	3361	701	337	<0.001**
	10-12 years	58.2%	3166	651	266	<0.001**
Spleen score	0	54.3%	4049	778	866	-
	1	65.9%	5695	1576	135	< 0.001**
	2	71.3%	7067	1138	224	< 0.001**
	3	66.7%	13675	1787	16	0.080
Hematocrit class	< 32%	62.1%	6583	1076	609	-
	32%-35%	55.3%	3587	688	360	<0.001**
	>35%	53.5%	2855	801	272	<0.002*

To best represent the distribution of malaria parasites in different classes of the population studied, three parameters are represented: prevalence of *P. falciparum* (%), arithmetic mean of parasite density (AMPD in parasites/ul), and geometric mean of parasite density (GMPD in parasites/ul). (n) Number of subjects in each class. (‡) p-value obtained by comparing GMPDs with the first row as the reference, using Wilcoxon tests. (*) Statistical significance was set at 0.05. (**) p-value less than 0.001.

More importantly, the GMPD was surprisingly high in the first dry season studied, similar to that found in the rainy season when considering all children (p = 0.934), and this was also true within all age classes taken separately. The GMPD from the first dry season was significantly higher than in the second dry season (p < 0.001 for all children) and in the youngest children only (aged 1-3y.), this difference was not significant (see Discussion section).

### Prevalence of fever and its associations with clinical data

Fever prevalence between seasons was first compared in univariate analyses between seasons using a low (37.5°C) or higher (38°C) threshold. Using the 37.5°C threshold, fever prevalence in the first dry season (12.0%, 95% CI: 9.8-14.7) was higher, but not significantly different from the rainy season (8.8%, 95% CI: 6.9-11.1, p = 0.062), but it was significantly higher than in the second dry season (6.6%, 95% CI: 4.9-8.7, p < 0.001). If the threshold was set at 38°C, fever prevalence was significantly higher in the rainy season (3.2%, 95% CI: 2.0-4.7) than in the second dry season (1.0%, 95% CI: 0.5-2.2, p < 0.02), but not significantly different from the first dry season (2.2%, 95% CI: 1.3-3.6, p = 0.353). In any case, the high prevalence of fever observed in the first dry season suggests that an outbreak of unknown origin could have occurred around that period of time.

When stratifying children into five classes of parasite density, there was a frequent, though not systematic, association between moderate or high parasite density (>2,000 p/μl or >20,000 p/μl) and fever: it is worthy of note that moderate parasite density (>2,000 p/μl) was not associated with fever during the first dry season, although a high GMPD was found (p = 0.089). Finally, very low (<200 p/μl) and low parasite density (<2,000 p/μl) was *never* associated with fever during *any* season. This observation is common when endemicity is high, raising some concern with regard to the usefulness of parasite detection in febrile children, especially if parasite density cannot be estimated due to physiological dynamics and technical limitations (RDTs do not allow an estimation of parasite density).

Although higher percentages of febrile children were observed among those with severe anaemia, when adjusting for age, the prevalence of fever (38°C or 37.5°C) was not significantly higher among children with severe anaemia compared to those without severe anaemia, during any of the three seasons studied. Therefore, age appeared as a confounding factor when measuring the association between anaemia and fever. The same pattern was observed for the association between splenomegaly and fever for a threshold set at 38°C, but not for a threshold set at 37.5°C: in this case, these two variables were linked during the rainy season (p < 0.002), the second dry season (p < 0.005), but not significantly during the first dry season (p = 0.073).

## Discussion

Although malaria is considered to be the main factor responsible for anaemia where *P. falciparum* is endemic, other factors can contribute to it (malnutrition, haemoglobinopathies, intestinal parasites, etc.). In the village where this study was carried out, previous analyses showed that these factors were present but at a level which cannot explain a significant effect beside falciparum malaria. Therefore, anaemia and/or levels of PCV can be used, at least at a population level, to detect a rise in malaria transmission and malaria disease.

If parasite density is a parameter to consider in a febrile child, it is not sufficient for diagnosing the malaria disease for two main reasons: first, even when malaria parasite discovery is concomitant with fever, it is not necessarily causal due to the high proportion of asymptomatic cases. For example, it has been shown that a certain number of patients living in a hyperendemic area carried bacterial infections [19]. Second, evidence of malaria and accurate counts are difficult to carry out, even when laboratory performance is considered as good. Parasite density can vary within a few hours from negative to positive and conversely in asymptomatic patients [20,21]. In contrast to parasite density, haematocrit is a more stable indicator whose variations occur within days, weeks or months.

Although these results were obtained in a series of three cross-sectional surveys, they highlight classical seasonal patterns of malaria indices: they show a seasonal rise of severe anaemia/moderate anaemia and splenomegaly during the high-transmission season, and found a strong association between anaemia and age, splenomegaly and moderate-to-high parasite density. This latter association, as well as the absence of link with low or very low parasite density confirms previous findings [22-25].

It should be noted that the observed important variations of malaria indices between seasons occurred while the prevalence of *P. falciparum* did not follow any significant change: in these particular epidemiological conditions (high transmission), there is a discrepancy between the presence of malaria parasites and malaria-related morbidity.

The fact that fever was associated only with a few of the malaria indices can be explained by the presence of other diseases, and by age being a confounding factor, the youngest children being the most affected by morbidity signs. It is to note however that low or very low parasite densities (below 2,000 parasites/μl) were never associated with fever, even if not adjusting for age, suggesting a high proportion of asymptomatic carriers within this range of parasite densities. This observation argues in favour of a diagnosis using indicators supplementary to the presence of parasites: the detection of *Plasmodium* in the blood appears as necessary, but not as sufficient to assert malaria as the origin of fever.

Interestingly, these data from two consecutive dry seasons show a striking difference in the prevalence of fever. In general, the observations of the first dry season appear unusual and those of the second dry season showed the more expected “low” values for the following indices: fever prevalence, parasite density, rate of severe anaemia and rate of splenic enlargement [8]. Two hypotheses can be considered with regard to the main origin of fever during the first dry season: 1- a transitory high transmission of malaria; or 2- any other outbreak of other infections, such as acute respiratory infections, measles.

The first hypothesis finds its stronger argument in the fact that the mean parasite density was particularly high, and as high as in the rainy season. Statistically, high parasite densities are associated with an increased risk of fever [26-28]*.* Therefore, the observation of high parasite densities during that first dry season could suggest that malaria transmission had abnormally increased, explaining a rise in clinical malaria cases. Nonetheless, parasite density cannot be seen as the unique risk factor: many studies attempted to estimate a cutoff parasite density supposed to trigger fever and clinical malaria (pyrogenic threshold) but there is no consensus around this theoretical value, which instead appears to vary with age and epidemiological conditions [29-34].

The second hypothesis - most febrile children suffered from an infection that is not malaria - is supported by several arguments: first, the observation that most fevers from this first dry season were below 38°C may suggest a disease other than malaria, as this latter is generally associated with higher body temperatures [26]. Second, in the first dry season, fever was not associated with moderate parasite density (>2,000p/μl) whatever the chosen threshold (37.5°C or 38°C), although this was the case for the other seasons studied. This may reflect the fact that even a high GMPD is not directly linked with the origin of fever. Third, the estimations of average daily precipitations from the periods preceding this survey do not argue in favour of an increase in malaria transmission during the first dry season. Fourth, the splenic rate and the rate of severe anaemia differed from those observed during the rainy season, when they increased with a two-fold magnitude, and fever was not associated with severe anaemia in any of the dry seasons. If malaria was the origin of fever during this first dry season, one could expect that these indices would have followed such a pattern: on the contrary, severe anaemia and splenomegaly were found to be low and their association –or absence of association- with fever were similar during both dry seasons. However, to give nuances to this fourth argument, the spectacular rise in severe anaemia and splenomegaly observed during a rainy season generally result from repeated infections over weeks or months, while the first hypothesis only supposes a transitory increase in malaria transmission. In addition, one should keep in mind that these two parameters –i.e. anaemia and splenic rates- are to be considered as *middle-term indices*, because they vary with a delay of several days or weeks after the malaria disease is declared, in contrast to parasite density or body temperature (several hours or days). Therefore, despite the fact that those parameters are expected to be more sensitive to variations in a low-transmission season (in this type of seasonal surveillance, changes in some malaria indices would be more easily detected if their baselines were low), it remains also possible that this survey happened at the very beginning of a malaria epidemic, when anaemia and splenomegaly had not yet increased significantly.

With the data available here, it is not possible to go further this discussion and choose one hypothesis or the other. In particular, the cure or relapse of symptoms in children within the days after treatment would have been useful to determine *in fine* the disease affecting each child. This work did not include such a follow-up (but made three case–control studies over several days, whose results are not discussed here); this may be needed in further studies. Nonetheless, these data show important seasonal changes in the pattern of malaria indices, reflecting classical or particular conditions in which *“stable”* indicators (such as PCV and splenomegaly) reflect much better the differences between high or low transmission seasons. The main argument to explain the difference between the two dry seasons is not based only on haematocrit or parasite density, but on a robust agreement between all the indices recorded in this study.

Although determining the origin of fever and the appropriate treatment will remain a probabilistic approach whatever the diagnosing strategy is, a better understanding of the malaria disease would help health workers by clarifying the most useful clinical signs with which to assess patients. Fever incidence sometimes suddenly increases in regions where malaria can be present, and relying only on the detection of parasites in patients can be misleading. Routine surveys of malaria indices over several seasons can usefully increase the comprehension of malaria with regard to regional conditions: in return, it could foster greater confidence in the identification of subsequent epidemics.

Moreover and possibly the most important, the knowledge of malaria endemicity in a given area is probably the best information to decide whether or not a systematic laboratory detection of malaria parasites in febrile children would provide a better “proof” of its *Plasmodium* origin [5].

Finally, these results are a strong argument to advocate the necessity, in any case, to analyse research and routine data on malaria separately for the dry and the wet seasons in areas where an important seasonal variation occurs in the transmission of malaria.

## Abbreviations

PCV: Packed cell volume (haematocrit); SA: Severe anaemia; MA: Moderate anaemia; GMPD: Geometric mean of parasite density; RDT: Rapid diagnostic test.

## Competing interests

The authors declare that they have no competing interests.

## Authors’ contributions

DLA performed data analysis and manuscript preparation. OKD and AM managed the teams for data collection; TS supervised the collection of clinical data. AR developed the concept and the design of the study. AR, BG, JF and SM directed the database analysis. All authors read and approved the final manuscript.

## Supplementary Material

Additional file 1 **Anaemia among different strata of children.** Mean haematocrit values (PCV), severe anaemia (SA) and moderate anaemia (MA) rates and odds ratios (OR) showing their associations with various variables in children. (n) Number of subjects in each class. (‡) p-value obtained by comparing values from different strata with the first line as the reference, using Wilcoxon tests (PCV means) or Chi-square tests (SA and MA rates). (*) Statistical significance was set at 0.05. (**) p-value less than 0.001. Click here for file
